# Thermally-assisted Magma Emplacement Explains Restless Calderas

**DOI:** 10.1038/s41598-017-08638-y

**Published:** 2017-08-11

**Authors:** Antonella Amoruso, Luca Crescentini, Massimo D’Antonio, Valerio Acocella

**Affiliations:** 1 Dipartimento di Chimica e Biologia, Università di Salerno, Fisciano, SA Italy; 2 Dipartimento di Fisica, Università di Salerno, Fisciano, SA Italy; 3 Dipartimento di Scienze della Terra, dell’Ambiente e delle Risorse, Università Federico II di Napoli, Napoli, NA Italy; 4 Dipartimento di Scienze, Università Roma Tre, Roma, Italy

## Abstract

Many calderas show repeated unrest over centuries. Though probably induced by magma, this unique behaviour is not understood and its dynamics remains elusive. To better understand these restless calderas, we interpret deformation data and build thermal models of Campi Flegrei caldera, Italy. Campi Flegrei experienced at least 4 major unrest episodes in the last decades. Our results indicate that the inflation and deflation of magmatic sources at the same location explain most deformation, at least since the build-up of the last 1538 AD eruption. However, such a repeated magma emplacement requires a persistently hot crust. Our thermal models show that this repeated emplacement was assisted by the thermal anomaly created by magma that was intruded at shallow depth ~3 ka before the last eruption. This may explain the persistence of the magmatic sources promoting the restless behaviour of the Campi Flegrei caldera; moreover, it explains the crystallization, re-melting and mixing among compositionally distinct magmas recorded in young volcanic rocks. Our model of thermally-assisted unrest may have a wider applicability, possibly explaining also the dynamics of other restless calderas.

## Introduction

Calderas are depressions related to the partial emptying of magmatic reservoirs, often associated with large eruptions. Calderas commonly experience unrest, testified by seismicity, ground deformation and degassing. Several calderas are restless over centuries, following repeated shallow magma emplacement. The forecast of any eruption and vent-opening site at these restless calderas is much more challenging than at any other type of volcano^1,2^. The restless behaviour may result from continuously-fed large and long-lived magmatic reservoirs. However, the detailed physico-chemical conditions promoting repeated unrest have been poorly investigated, remaining elusive and hindering our capability to forecast eruptions.

**Figure 1:**
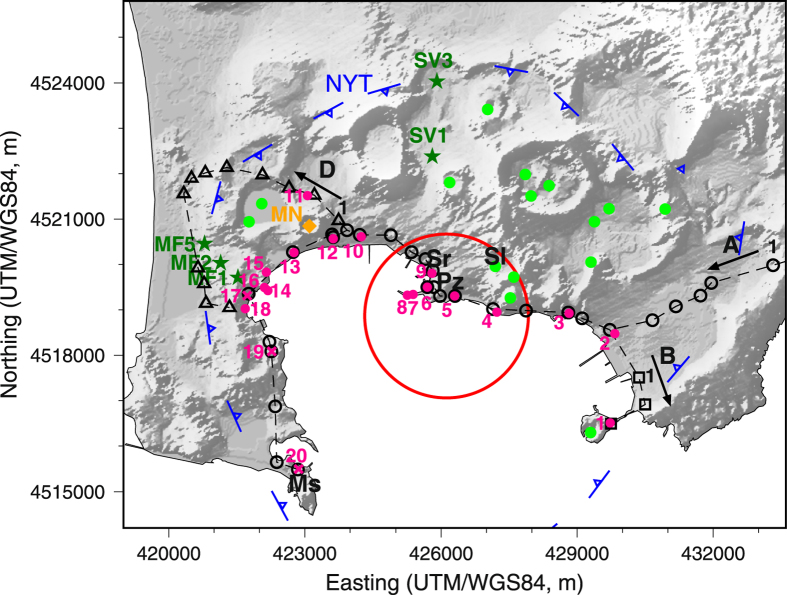
Campi Flegrei caldera. Green dots, third epoch vents; MN, Monte Nuovo (1538 AD eruption); Pz, Pozzuoli; Ms, Miseno; Sr, Serapeo; Sl, Solfatara; black symbols, levelling benchmarks; magenta dots, 1400–1536 data sites^11^; dark green stars, AGIP boreholes^28^; red circle, surface projection of the ~3600 m deep sill used in this work. A, B and D and related dashed lines indicate levelling routes in Fig. 2. (Map generated using GMT v. 4.5.12, http://gmt.soest.hawaii.edu/; relief from a 20-m Digital Elevation Map freely provided by the Italian Istituto Superiore per la Protezione e la Ricerca Ambientale, http://www.sinanet.isprambiente.it/it/sia-ispra/download-mais/dem20/view).

To better understand restless calderas, we consider the recent history of Campi Flegrei, one of the best-known, yet most dangerous calderas, lying to the west of Naples and restless since the 1950s at least. Several tens of eruptions, divided in three epochs, and resurgence of the caldera centre (Pozzuoli area – Fig. 1) accompanied the last 15 ka^3,4^. The 24 eruptions of the last epoch (~5.6 to ~3.7 ka^5^) totalled ~2 km^3^ of dense rock equivalent (V_DRE_) erupted magma^6^. After ~3 ka of quiescence and >1 ka of subsidence from the Roman period to the Middle Ages, in the 1400–1536 period increasing seismicity and uplift preceded the last 1538 AD eruption, Monte Nuovo (MN in Fig. 1), followed by deflation. Four major unrest episodes occurred between 1950–1952, 1969–1972, 1982–1984 and 2005–Present, with magma-driven uplift at Pozzuoli of ~0.7, ~1.7, ~1.8 and ~0.4 m, respectively^7–11^. In particular, the post-1982 ground displacement has consistently exhibited the same pattern during uplift and subsidence periods, being satisfied by the inflation and deflation of one or more ~3600 m wide and ~3600 m deep magmatic sill(s)^12,13^. Other studies underline the important role of magmatic gases on the deformation^14^, also heating the hydrothermal fluids and rocks^15^. These and other petrological studies^16,17^ concur in delineating the commonly accepted general architecture of the magmatic plumbing system below Campi Flegrei; this consists of an ~8 km deep main oblate reservoir, where mantle-derived primitive magmas reside undergoing differentiation and from which more differentiated magmas rise toward smaller oblate reservoirs, at 3–4 km depth. This magmatic model is also consistent with the eruptions of more primitive magmas (mainly through regional fracture systems^18^) and the gas release from primitive magma in the deeper reservoir, feeding the shallower reservoirs^14^.

## Results

**Figure 2:**
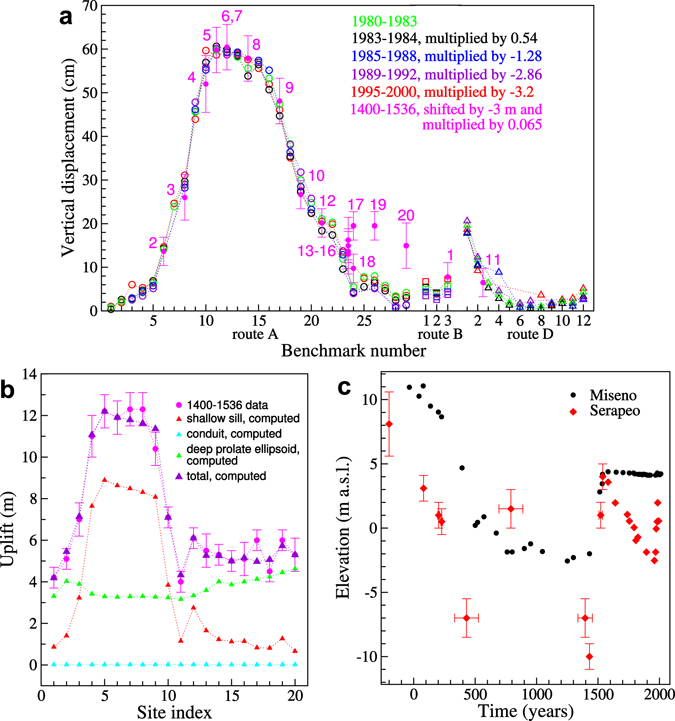
Vertical displacements in the last 2 ka. (**a**) Uplifts for different time periods along levelling benchmarks (symbols as in Fig. 1); magenta dots, 1400–1536 vertical displacement data and error bars^11^ after subtracting a uniform 3 m upheaval. (**b**) Comparisons between 1400–1536 data and computations; site indexes as annotations in Fig. 1 and (**a**). (**c**) Deformation in the caldera centre (red diamonds, Serapeo) against caldera rim (black circles, Miseno)^47^.

Here we first test whether the persistent displacement pattern observed from 1982 to Present at Campi Flegrei occurred also earlier than 1980, analysing available ground displacement data before the last eruption. To this aim, we analyse the 1400–1536 deformation pattern^11^. In this period, an uplift of a fe﻿wmetres was observed in the peripheral areas of the caldera (e.g., sites 17, 19, 20; Figs 1 and 2a,b). In these same sites there has been no uplift during the unrest episodes of the last decades. To test whether there is any similarity in the deformation pattern of the last decades and during 1400–1536, we removed 3 m of uplift from the pre-Monte Nuovo eruption pattern. After removal of this signal from the 1400–1536 deformation, we observed the same deformation pattern (see Fig. 2a) as in the recent unrest episodes, even though with different absolute uplift values. To scale these uplifts, we considered the ratio between the maximum uplift (0.6 m) during 1980–1983 (when detailed geodetic and gravimetric data were acquired) and that before the Monte Nuovo eruption (9.3 m), giving a scaling factor of 0.6/9.3, that is 0.065 (Fig. 2a).

**Figure 3:**
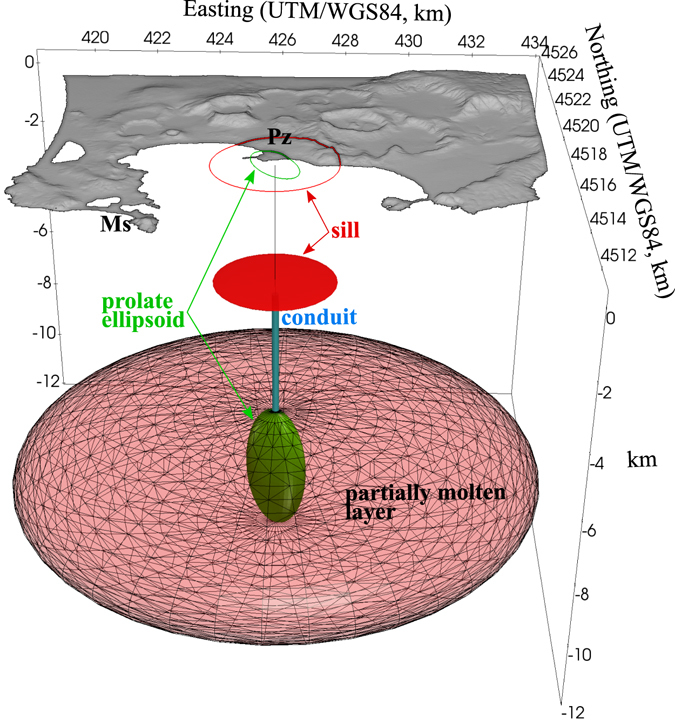
Schematized magmatic system of Campi Flegrei caldera. Sources reconstructed from surface deformation data in the last ~700 years.

This original re-interpretation of previous data indicates that the 1400–1536 deformation pattern consisted of a quite uniform upheaval of ~3 m and a more central uplift, similar to what recently observed. The former signal can be explained by the pressurization of a prolate ellipsoidal source at ~8 to 11 km depth, which may represent the feeder conduit system of the geophysically detected wider subhorizontal magma reservoir^19^ (Figs 2b and 3; see Methods). The latter signal can be explained by an oblate ellipsoidal, or sill-like, source at ~3.5 km depth (Fig. 2b). The shallower sill-like and deeper prolate ellipsoidal sources are thus supported by the different uplift behaviour between the caldera centre (e.g., Serapeo, Sr; Fig. 1, affected by both the sill and the prolate sources), and the caldera rim (e.g., Miseno, Ms; Fig. 2c, mainly affected by the prolate source). However, any narrow conduit connecting these sources remains geodetically undetected.

Figure 2a also shows that the same pattern is shared by both inflation and deflation episodes during the last decades. More generally, surface deformation is always satisfied by inflation and deflation of a same pressurized shallow (~3.5 km depth) source during the last decades^12,13^. Superficial deformation from this source, normalized to the maximum absolute vertical displacement, is what we define “persistent displacement pattern”. The consistency of both the inflation and deflation deformation patterns cannot be reproduced through hydrothermal sources, which produce transient variations^20^. This underlines the importance of magma dynamics in any recent surface deformation at Campi Flegrei.

The longer-term uplifted area during resurgence broadly matches the sill-like surface projection^11^, suggesting the persistence and activity of the same sill-like source(s) over the last 5 ka.

To define the evolution and consequences of the relict thermal anomaly, we compute the temperature variations beneath Campi Flegrei through an axisymmetric Finite Elements Model, using COMSOL Multiphysics v5.2.

The computational domain is a cylinder, 50 km in radius and 50 km in height. The permanent deeper source is schematized as a horizontal oblate spheroid whose equatorial radius is 5500 m, polar semi-axis is 500 m and depth is 8000 m; its temperature is kept constant at 1050 °C. The bottom boundary condition is a uniform constant inward heat flux equal to 0.06 W m^−2^ (see ref.^21^). The temperature of the top boundary of the domain is fixed at 15 °C and the lateral surface is thermally isolated.

We assume a purely conductive behaviour, which will be justified a posteriori on the basis of the results (see Methods); thermal conductivity is assumed 1 W K^−1^ m^−1^ down to 1500 m depth, because of the occurrence of tuffaceous rocks, and 2 W K^−1^ m^−1^ underneath^22^.

We superimpose the assumed 3600 m deep thermal anomaly at 3.7 ka on the stationary temperature distribution obtained as before, and allow the temperature distribution to evolve over time. The assumed thermal anomaly is schematized as a hot (750 °C, below the solidus temperature of Campi Flegrei trachytic magmas^23,24^) oblate region, whose equatorial and polar radii are 3000 and 300 m respectively, and with inferred volume ~5 times larger than the third epoch magma V_DRE_ (see Introduction section).

**Figure 4:**
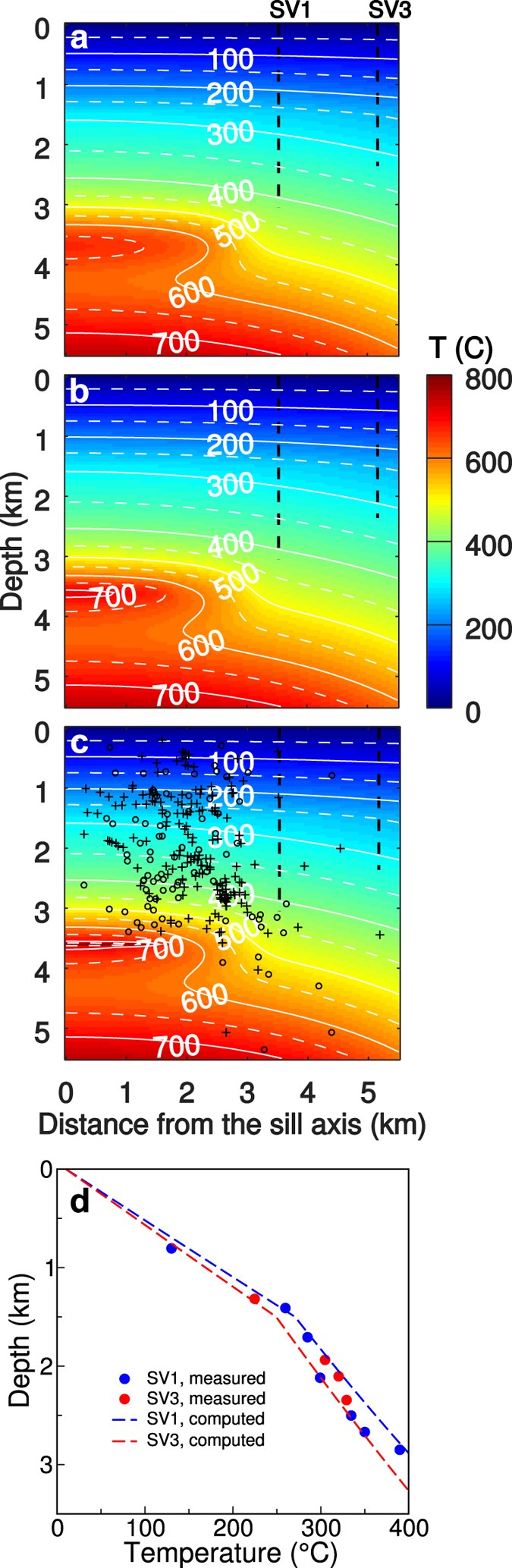
Thermal model. (**a**) Computed thermal section before the 1538 eruption; black dashed lines, SV1 and SV3 boreholes. (**b**) Same as (**a**) but around 1950. (**c**) Same as (**b**) but shortly before 1980; black circles, well located (rms < 0.05) earthquakes from 1982 to 2000^48^; black pluses, well located earthquakes from 2000 to 2014^48^. (**d**) Dots, SV1 and SV3 temperature data; dashed lines, computed temperature profile at SV1 and SV3 around the half of the 20^th^ century.

Our thermal models show that after 3000 years, i.e. shortly before the 1538 eruption, the temperature at the centre of the thermal anomaly is still >650 °C (Fig. 4a). The ~0.2 km^3^ sill volume change before the 1538 eruption is consistent with an intrusion, 1800 m and 15 m in equatorial and polar radii respectively, at 3600 m depth. This implies an addition of hot material to the shallow magmatic system with initial temperature of 950 °C, consistently with geothermometry estimates and high T-P experiments on Campi Flegrei trachytes^23–25^. We account for the latent heat released during cooling and solidification using an effective magma specific heat, which includes both sensible and latent heats^26^. Our models show that after 400 years (half of the 20^th^ century) the temperature of such a sill is still >700 °C, close to the solidus temperature (Fig. 4b). We follow a similar approach for computing the temperature distribution evolution after the magma intrusions occurred in the seventies and eighties, adding a new hot (950 °C) sill to the temperature distribution computed immediately before each intrusion, as initial condition for subsequent thermal evolution. For each intrusion, the sill radius is 1800 m and the sill thickness is estimated from ground displacements on a case-by-case basis.

Several tests show that increasing depth and thickness of the permanent deeper source by a couple of km does not change model results significantly. Theoretical calculations suggest that the magma can avoid freezing and develop a sill only if the country rock temperature is close to the magma solidus temperature, and the horizontal thermal gradient is very small^27^. Therefore, the lateral migration of the sill(s) responsible for the recent unrest episodes was mainly thermally controlled and confined by the stronger radial temperature gradients, persistently occurring ~2 km from the sill axis (Fig. 4c). This lateral confinement also explains the consistency of the deformation pattern and its sources, which remain nearly constant through time. During spreading, the sill radius increases over time and so does the width of the ground displacement pattern; however, this signature is practically undetectable by the existing monitoring techniques until the sill reaches the final radius and starts to thicken (see Methods). Any viscoelastic behaviour of the crust does not affect the reconstructed deformation pattern appreciably (see Methods). Below the caldera centre, our reconstructed thermal distribution is strongly affected by the shallow thermal anomaly (Fig. 4). Conversely, towards the caldera periphery, the distribution is primarily affected by the background heat flux and the deeper subhorizontal reservoir, consistently with temperature data from two deep boreholes^28^ (SV1 and SV3; Figs 1 and 4d); these boreholes, unlike others along the most fractured caldera rim (MF1, MF2, MF5; Fig. 1) are inferred to better represent the general gradient of the area^22^. Our reconstructed temperature distribution also shows how the well-located earthquakes from 1982 to 2014 (see Methods) cluster in regions with T < 600 °C (Fig. 4c), supporting the thermal control on the distribution of seismicity.

## Discussion and Conclusions

The presence of a persistent shallow magmatic reservoir is also supported by geophysical (coda wave attenuation imaging^29^ and gravity^7^) data. Moreover, petrological data suggest that mafic magmas rise from the mantle up to a 8–10 km deep crustal level beneath Campi Flegrei, where they stagnate and differentiate; from there, silicic magmas rise towards 3–4 km deep crustal reservoirs^17^. At that depth, repetitive crystallization and re-melting events occur, as testified by complex growth and resorption textures in sanidine phenocrysts^25^. Those events are likely related to repetitive mixing among compositionally distinct magmas rising from the deeper reservoir and interacting with those resting in the shallower reservoir. This has been documented for several Campi Flegrei eruptions occurred in the past 5 ka^17,30^. Thus, also petrological data support the hypothesis of a stable 3–4 km deep reservoir responsible for a thermal anomaly lasting at least since the last epoch (3.7 ka).

The proposed thermal model is in general consistent with previous models explaining the thermal evolution of volcanic environments through repetitive intrusions^31^.

In addition, our model explains the systematic partial recovery of the ground deformation of the last ~2 ka, a poorly investigated feature of Campi Flegrei^32^. In the last 5 ka, magma mostly had a water content of 2–5 wt.% and CO_2_ content of 50–1100 ppm^25,30^. Magmatic water and CO_2_ are released as supercritical fluids during magma solidification and may migrate towards shallower depths. Supercritical water density is ~200 kg/m^3^ at 3.6 km depth and magma solidus temperature (P ~ 80 MPa and T ~ 750 °C; see Methods). Neglecting the small contribution of CO_2_, the volume fraction of supercritical water in the magmatic source after full magma solidification (~700°C) is 25–30%. The release of this supercritical water justifies the years-long, and contributes to the centuries-long, volume decrease of the source during the deflation after the major uplifts^8,12^. This release is supported by seismic tomography data^19^, showing fractured thermo-metamorphic rocks hosting supercritical fluids at 3–4 km depth, and geochemical data, correlating degassing with minor uplift episodes on shorter timescales^33^.

As for the possible thermal consequences of this fluid release, thermodynamic transformations of the fluids due to decompression and cooling heat the surrounding rock along the migration paths, thus decreasing the local conductive heat flux from below. If such a heat was released uniformly inside the caldera volume from the sill depth up to the surface, it would induce only a negligible perturbation in temperature (~1/10 °C). To justify this number, we note that the heat released by the fluids (assumed ~3 wt.%) set free by one kilogram of magma is on the order of 2000 J kg^−1^ MPa^−1^ for each 1 MPa decrease in pressure by the fluids^15^, i.e. about 1.6 × 10^5^ J kg^−1^ along the whole path from the sill to the surface (~80 MPa). As regards the 1982–1984 major uplift^12,13^, the sill volume change was about 5 × 10^7^ m^3^, thus the magma mass was about 1.2 × 10^11^ kg and the total heat released by the fluids set free by the magma was about 2 × 10^16^ J. If fluid migration was homogeneous in the rock volume above the sill (rock volume and mass about 4.5 × 10^10^ m^3^ and 10^14^ kg, respectively) and heat release was immediate, this large amount of heat would cause a temporary mean temperature change of 0.2 K for a rock heat capacity of about 840 J kg^−1^ K^−1^. However, fluid migration and heating are probably localized in the small region below Solfatara and other minor fumaroles. This increases the local rock temperature change, but decreases the affected portion of the thermal model. Thus, heat released by thermodynamic transformations of the fluids due to decompression and cooling does not affect our model significantly.

Our computed superficial conductive heat flux inside a circle 7000 m in radius (roughly corresponding to the caldera size) is ~25 MW at present. If we double the thermal conductivity at all depths, the heat flux becomes about 47 MW, without affecting the computed temperature distribution significantly. Moreover, both the superficial heat flux and the temperature distribution are barely affected by the values adopted for the bottom-boundary inward heat flux. The superficial conductive heat flux cannot be compared to the thermal energy release at Solfatara (~100 MW^34^), which is mainly due to thermodynamic transformations of the magmatic fluids. Moreover, at least part of the gas emitted at Solfatara may originate from a primitive (i.e., deep) magma^14,35^.

We conclude that the heat produced by the magmatic intrusions during the last epoch and before the 1538 eruption thermally assisted the repeated emplacement of the later intrusions responsible for the recent unrest episodes. The restless behaviour of Campi Flegrei thus appears self-sustained by the magma intruded earlier, which promoted the intrusion of the shallow sills. These sills are fed through the prolate ellipsoid at *~*8 km depth. We cannot exclude that the magma that will feed any future eruption might still derive from that magma which already pressurized the prolate source before 1538. However, any future eruption should be expected to be fed by fresh, mafic hot magma arriving from the mantle, first inflating the deeper prolate source. This possibility should be detected through “anomalous” (i.e., not consistent with the established deformation pattern of the last decades) ground displacements along the caldera rim, whose sites are mainly affected by the prolate source. Therefore, we emphasize to accurately monitor surface deformation along the caldera periphery (e.g., at Miseno) to better forecast any eruption.

Finally, available information at other calderas highlights similarities to Campi Flegrei, in the pattern and cause of unrest. All monitored restless calderas have either geodetically (Yellowstone, Aira Iwo-Jima, Askja, Fernandina and, partly, Long Valley^2^) or geophysically (Rabaul, Okmok^36,37^) detected sill-like intrusions inducing repeated unrest. Some calderas (Yellowstone, Long Valley) also show stable deformation pattern, where inflation insists on and mimics the resurgence uplift^2^. The common existence of sill-like sources, also responsible for stable deformation patterns, in restless calderas suggests close similarities to Campi Flegrei. This suggests a wider applicability of our model of thermally-assisted sill emplacement, to be tested by future studies to better understand the dynamics of restless calderas and their eruptive potential.

## Methods

### Parameters of the 1400–1536 AD deformation source(s)

The persistent sill source was originally schematized as a Pressurized Triaxial Ellipsoid (PTE)^12,13^. Here we model the deeper prolate source as a triaxial ellipsoid, and the persistent sill source as an oblate spheroid (Figs 1 and 3).

Physical properties of the hosting layered medium are obtained from seismic tomography^7^. We compute ground displacements from both sources using the analytical quadrupole approximation (seven moment-tensor model) for a finite uniformly-pressurized ellipsoidal cavity^38^. Volume changes are computed based on ref.^39^. We fix the position of the centre of the oblate spheroid (426119 E 4518866 N, UTM WGS84 33N; depth 3600 m), the vertical orientation of its symmetry axis, the coordinates of the centre of the deeper source (same as for the oblate spheroid), and the vertical orientation of one of its axes. We also fix the equatorial radius of the oblate spheroid at 1800 m, which is the average of the PTE horizontal semi-axis lengths estimated by fitting recent ground deformation (Probability Density Functions of PTE parameters in ref.^13^). The orientation of the deeper source horizontal axes, the depth to its centre, the lengths of the PTE minor axis and all the deeper source axes, and the volume changes of both sources are obtained from least absolute deviation fitting of uplift data. We find that the oblate spheroid volume change is ~0.2 km^3^, the longest axis of the deeper source (prolate ellipsoid) is vertical, its depth is 8 to 11 km, and its volume change is 3 to 4 km^3^, depending on depth. The ratio between the two horizontal axes of the prolate ellipsoid is constrained to ~0.5 by the SW data sites; the ratio between the major horizontal axis and the vertical axis is <0.6. Computed displacements in Fig. 2b refer to the following numerical case. Oblate spheroid: volume change, 0.21 km^3^. Prolate ellipsoid: depth to the centre, 10 km; volume change, 3.9 km^3^; vertical semi-axis, 2000 m; horizontal semi-axes, 1000 m and 550 m; azimuth of the major horizontal axis, 60°. Cylindrical conduit: volume change, 0.05 km^3^.

The only unmatched uplift is at site 17, very close to four well-fitted sites (14, 15, 16 and 18) and is thus an outlier (Fig. 2b).

### Thermal model

In order to test the assumption of a purely conductive thermal model, we consider the problem of a horizontal layer of a porous medium uniformly heated from below. Natural convection occurs if the Rayleigh-Darcy number Ra = ρ^2^βgKHc_p_ΔT/(kμ) is larger than ~40^40^; here ρ is fluid density, g is gravity, β is fluid thermal expansion coefficient, K is rock permeability, H is layer thickness, c_p_ is fluid specific heat at constant pressure, ΔT is temperature difference between the top and bottom surfaces, k is thermal conductivity of the saturated rock, and μ fluid dynamic viscosity. Since Ra increases with H and ΔT, we consider the unlikely (because of layered heterogeneities) scenario producing the highest Ra, i.e. H = 3600 m and ΔT = 700 K. We use k = 2 J m^−1^ K^−1^ and, considering high-density supercritical water, ρ = 600 kg m^−3^, β = 3 × 10^−3^ K^−1^, c_p_ = 5 × 10^3^ J K^−1^ kg^−1^, and μ = 10^−4^ Pa s; rock permeability from the deep AGIP wells is generally on the order of 10^−17^ m^2^ (see refs^41,^ ^42^). The obtained Ra ~ 7 justifies the purely conductive model used.

### Sill spreading modelling

At first, we check for the effects of the free surface on the sill shape. We consider a uniformly pressurized, 3600 m deep, circular sill embedded in a homogeneous elastic half-space whose Poisson ratio ν is 0.25; the sill radius R ranges 1000 m to 16000 m. We compute the sill face displacements using the COMSOL Multiphysics v5.2 with a 2D axisymmetric domain. Results are shown in Supplementary Fig. S1: the effects of the free surface are null for R up to 2000 m and significant for R > 4000 m. Here R < 2000 m; thus, we can model the sill as the spreading of a penny-shaped fluid-driven fracture in an impermeable infinite medium^43^. Spreading is controlled by what occurs at the fracture tip, e.g. a possible lag between the fluid magma edge and the tip. Magma viscosity μ for a trachytic magma with water content >2 wt.% ranges^44^ from 10^3^ to 10^6 ^Pa s and the injection rate Q for the recent uplifts ranges^12,13^ from 0.05 to 0.5 m^3^/s. Under these conditions, it can be shown that spreading mostly occurs in a regime where the lag is much smaller than the sill radius or absent and the effects of the rock toughness are negligible (self-similar M-solution^43^). It follows that the sill spreading time t_R_, i.e. time required for the sill to reach the radius R, is:

1 $${t}_{R}={(\frac{R}{\gamma })}^{9/4}{(\frac{12\mu (1-{\nu }^{2})}{E{Q}^{3}})}^{1/4}$$

where γ = 0.6955 and E is the rock Young’s modulus, which is ~30 GPa at 3600 m depth^12^; thus, t_R_ ranges weeks to months for recent CF uplifts.

We also compute the time history of ground displacements generated during the sill spreading phase for intermediate radii a ranging from 200 to 1800 m, using two injection rates (Q_1_ = 0.05 m^3^/s; Q_2_ = 0.5 m^3^/s) and two magma viscosities (μ_1_ = 10^4^ Pa s; μ_2_ = 10^5^ Pa s); sill depth is 3600 m (Supplementary Fig. S2a). Temporal changes of the displacement pattern are detectable only if the displacement detection limit of the monitoring network is much smaller than the standard deviation of the differences between ground displacements for a <1800 m and properly scaled-down ground displacements for a = R = 1800 m.

This standard deviation is shown in Supplementary Fig. S2b for an ideal measurement net, covering the whole deformed area; it demonstrates that, even in this ideal case, changes in the ground displacement pattern during the spreading phase are undetectable through existing monitoring techniques.

### Viscoelastic relaxation

We use the COMSOL Multiphysics v5.2 and the Structural Mechanics Module with a 2D axisymmetric domain. We model the behaviour of the viscoelastic material using the Standard Linear Solid model, consisting of two systems in parallel, the first containing a spring and dashpot in series, the other containing only a spring. To get clues on the viscosity relaxation effects on ground displacements, we use the same rigidity (7.5 GPa) for both springs, and compute the viscosity η of the dashpot through the simple Arrhenius formulation

2 $$\eta ={A}_{d}\exp (\frac{{A}_{E}}{RT})$$

where A_d_ is the Dorn parameter, A_E_ the activation energy, R the gas constant, and T absolute temperature. We use^45^ A_d_ = 10^9^ Pa s and A_E_ = 120 kJ mol^−1^ (Supplementary Fig. S3a); changing those values affects the characteristic time of the relaxation process, not its main features. The instantaneous Poisson ratio of the medium is 0.25. At first we consider a 3600 m deep, pressurized circular sill with 1800 m radius, a 10 m polar axis, and a 65 MPa overpressure. This overpressure is relatively high, as we refer to the volume variation related to the surface uplift. This volume, for a pressurized sill in a homogeneous half-space, depends upon the Poisson’s ratio ν, quite constrained (ν ~ 0.25)^21^. The translation of this volume variation into an overpressure requires proper knowledge of the medium Young’s modulus and sill radius, both closely controlling the resulting overpressure^39^. Therefore, this overpressure is an indirect outcome, reflecting our limited knowledge of the elastic properties of the crust and size of the source. We keep the pressure constant over time, and compute ground and sill wall displacements (Supplementary Fig. S3b). Then, we keep the previously computed instantaneous displacement of the sill walls constant over time, and calculate ground displacements (Supplementary Fig. S2c).

We obtain that viscoelastic behaviour of the crust does not affect the reconstructed deformation pattern. In fact, for both a sill with constant overpressure and constant volume change, we find that the ground displacement pattern is nearly constant over time, although displacements may vary by ~10%.

### Supercritical fluids

We compute the density ρ_w_ of supercritical water at P = 80 MPa and T = 1023.15 K (750 °C) using the on-line tool http://people.ds.cam.ac.uk/pjb10/thermo/pure.html, based on the Peng-Robinson two-constant equation of state^46^. The volume fraction of supercritical water inside the solidified magma fraction is given by

3 $${\phi }_{w}=\frac{{w}_{w}/{\rho }_{w}}{(1-{w}_{w})/{\rho }_{m}+{w}_{w}/{\rho }_{w}}$$

where *w*_*w*_ is the water percentage by mass and ρ_m_ is the density of the solidified magma fraction. Since ρ_w_ = 200 kg m^−3^, we get φ_w_ = 0.28 for ρ_m_ = 2500 kg m^−3^ and *w*_*w*_ = 0.03.

## Electronic supplementary material

Supplementary Information
