# Supplementary material for: Thermally-assisted Magma Emplacement Explains Restless Calderas
Source: Sci Rep. 2017 Aug 11;7:7948. doi: 10.1038/s41598-017-08638-y (PMC5554218; doi:10.1038/s41598-017-08638-y)
Supplement: Supplementary file 1 — Supplementary Information [file 41598_2017_8638_MOESM1_ESM.pdf]

SUPPLEMENTARY INFORMATION

Thermally-assisted Magma Emplacement Explains Restless Calderas

A. Amoruso[1,*], L. Crescentini[2], M. D'Antonio[3], V. Acocella[4]

[1]Dipartimento di Chimica e Biologia, Università di Salerno, Italy.

[2]Dipartimento di Fisica, Università di Salerno, Italy.

[3]Dipartimento di Scienze della Terra, dell'Ambiente e delle Risorse, Università Federico II di Napoli, Italy.

[4]Dipartimento di Scienze Roma Tre, Roma, Italy.

***Corresponding author (aamoruso@unisa.it).**

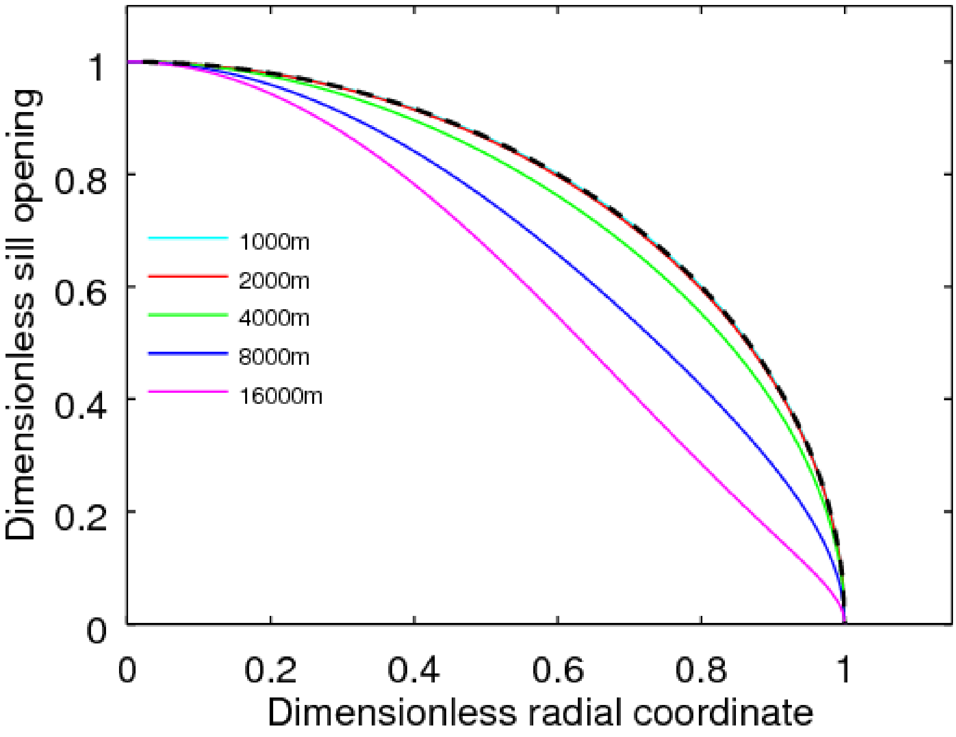

Supplementary Figure S1. Equilibrium shape of a uniformly pressurized, 3600 m deep, circular sill embedded in a homogeneous elastic half-space whose Poisson ratio ν is 0.25; the sill radius R ranges 1000 m to 16000 m. Solid lines, dimensionless sill opening (opening divided by its value at the sill axis) vs. dimensionless radial coordinate (distance from the sill axis divided by the sill radius). Dashed black line, analytical solution for a circular sill embedded in an infinite homogeneous medium[38].

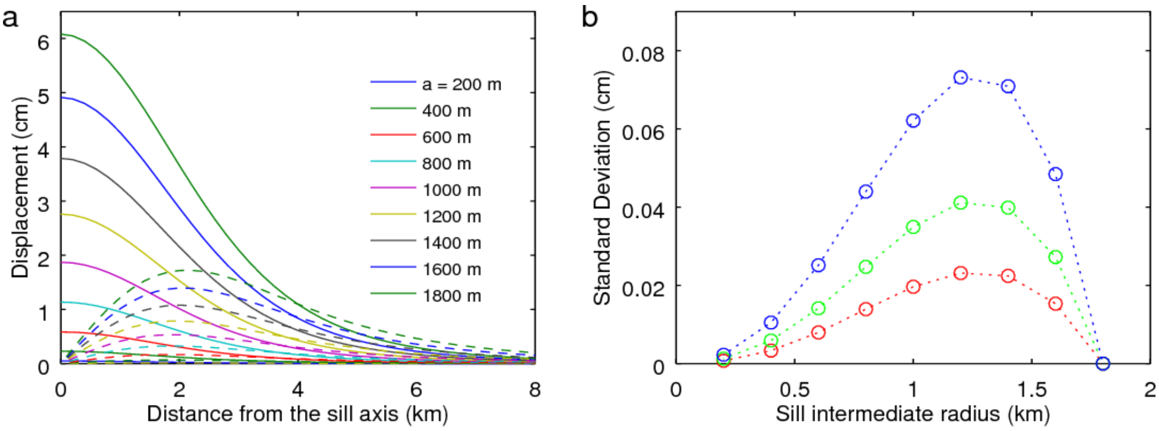

Supplementary Figure S2. Ground displacements during the spreading phase, up to 1800 m in radius, of a 3600 m deep, circular sill embedded in a homogeneous elastic half-space whose Poisson ratio ν is 0.25. **a**, vertical (solid lines) and radial (dashed lines) displacements for Q = 0.05 m^3/s and μ = 10^5 Pa s (or, equivalently, Q = 0.5 m^3/s and μ = 10^4 Pa s). **b**, standard deviation of the difference between ground displacements for the sill intermediate radii as in **a** and the best-fit scaled-down ground displacements for a = 1800 m; blue line, Q = 0.5 m^3/s and μ = 10^5 Pa s; green line, Q = 0.05 m^3/s and μ = 10^5 Pa s (or, equivalently, Q = 0.5 m^3/s and μ = 10^4 Pa s); red line Q = 0.05 m^3/s and μ = 10^4 Pa s.

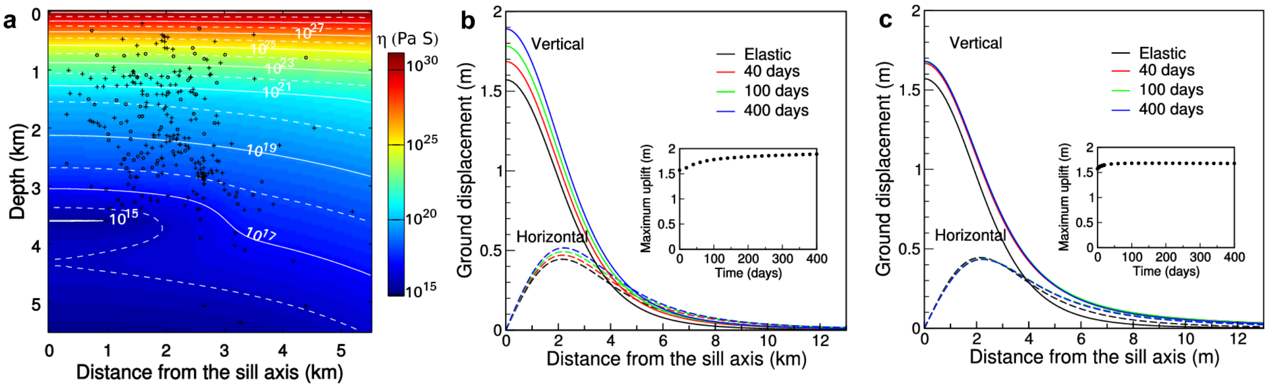

Supplementary Figure S3. Viscoelastic ground deformation. **a**, Computed vertical section of rock viscosity; white lines, logarithmic contours in Pa s; black circles, projections onto the section of well located (rms < 0.05) seismic events from 1982 to 2000[48]; black pluses, projections onto the section of well-located seismic events from 2000 to 2014[48]. **b**, Ground displacements due to the viscoelastic relaxation for a sill with constant overpressure; solid lines, vertical displacements; dashed lines, horizontal displacements; inset, maximum vertical displacement changes over time. **c**, Same as **b**, but for a sill with constant volume change.
